# Potential role of HIF-1-responsive microRNA210/HIF3 axis on gemcitabine resistance in cholangiocarcinoma cells

**DOI:** 10.1371/journal.pone.0199827

**Published:** 2018-06-28

**Authors:** Runglawan Silakit, Yingpinyapat Kitirat, Suyanee Thongchot, Watcharin Loilome, Anchalee Techasen, Piti Ungarreevittaya, Narong Khuntikeo, Puangrat Yongvanit, Ji Hye Yang, Nam Hee Kim, Jong In Yook, Nisana Namwat

**Affiliations:** 1 Department of Biochemistry, Faculty of Medicine, Khon Kaen University, Khon Kaen, Thailand; 2 Cholangiocarcinoma Research Institute, Khon Kaen University, Khon Kaen, Thailand; 3 Faculty of Associated Medical Sciences, Khon Kaen University, Khon Kaen, Thailand; 4 Department of Pathology, Faculty of Medicine, Khon Kaen University, Khon Kaen, Thailand; 5 Department of Surgery, Faculty of Medicine, Khon Kaen University, Khon Kaen, Thailand; 6 Department of Oral Pathology, Oral Cancer Research Institute, College of Dentistry, Yonsei University, Seoul, Korea; IRCCS-Policlinico San Donato, ITALY

## Abstract

MicroRNA-210 (miR-210) is a robust target for hypoxia-inducible factor, and its overexpression has been detected in a variety of solid tumors. However, the role of miR-210 in the development, progression and response to therapy in cholangiocarcinoma (CCA) remains undefined. We report here that high miR-210 expression was significantly correlated with the shorter survival of CCA patients. Overexpression of miR-210 inhibited CCA cell proliferation at the G2/M phase and reduced the gemcitabine sensitivity in CCA cells under CoCl_2_-induced pseudohypoxia. Concomitantly, inhibition of endogenous miR-210 activity using miRNA sponges increased cell proliferation under CoCl_2_-induced pseudohypoxia, resulting in an increase in gemcitabine sensitivity in CCA cells. We showed that HIF-3α, a negative controller of HIF-1α, was a target of miR-210 constituting a feed-forward hypoxic regulatory loop. Our data suggest an important role of miR-210 in sustaining HIF-1α activity *via* the suppression of HIF-3α, regulating cell growth and chemotherapeutic drug resistance in CCA.

## Introduction

Cholangiocarcinoma (CCA) is a cancer arising from the epithelial cells lining the intrahepatic and extrahepatic bile ducts caused by injury, inflammation and repair of the bile duct [1,2]. CCA is rare in most countries but has a high incidence in Southeast Asian countries bordering the Mekong River, especially Thailand [3]. Although surgical resection represents the best curative therapy [4], most patients present with advanced stage tumors that are incurable, allowing only palliative treatment. The only ways to control the disease and improve the patient’s quality of life are chemotherapy and radiation therapy [5,6]. Thus, understanding the molecular targets involved in the response to chemotherapy in CCA might improve the effectiveness of the therapies, as well as helping to establish new therapeutic strategies.

Hypoxia is a key component in the tumor microenvironments and represents a well-documented cause of therapeutic failure in solid tumors. Tumor cells survive under hypoxic conditions by controlling transcriptional and post-transcriptional events [7]. This response is mainly facilitated through hypoxia-inducible factor (HIF), a basic helix-loop-helix-PAS domain transcription factor composed of α- and β-subunits. To date, three structurally intimately related α-subunits, HIF-1α, HIF-2α, and HIF-3α, have been identified [8]. HIF-1α and HIF-2α contribute to tumor progression, whereas HIF-3α is a negative controller of HIF-1α [9,10], while the role of HIF-3a on the endogenous feedback regulatory loop under hypoxia is not well determined yet. To stabilize the HIF-1α - dependent hypoxic condition *in vitro*, cobalt chloride (CoCl_2_) has been widely used to establish a pseudohypoxic condition [11–13].

Over the past several years, various hypoxia-regulated microRNAs (miRNAs) have been identified. Among these specific sets of miRNAs induced by hypoxia, miR-210 is the most consistently and robustly upregulated [14]. The expression of miR-210 is regulated by HIF-1α in a variety of tumor types through binding at the hypoxia responsive element (HRE) [15], and its overexpression has been detected in most solid tumors, its presence being negatively correlated with the clinical outcome [16,17]. To date, several miR-210 targets have been identified that are involved in controlling cell metabolism, angiogenesis, DNA repair, cell survival and apoptosis [18].

Previous studies of CCA reveal that the hypoxic state induces an aggressive phenotype of CCA cells, controls cancer cell proliferation and enhances CCA metastasis, leading to a poor prognosis for CCA patients [19–21]. However, the role of miR-210 in regulating CCA progression and chemotherapeutic drug sensitivity in CCA cells under the hypoxic condition remains undefined. Therefore, the main objective of this study was to investigate the oncogenic roles of miR-210 on the hypoxia regulatory loop that contributes to a poor prognosis for CCA patients and drug resistance in CCA cells.

## Materials and methods

### Patient samples

Thirty-eight frozen intrahepatic CCA specimens and 30 cases of non-tumorous tissues of patients, collected from 2004–2010, were obtained from the specimen bank of the Cholangiocarcinoma Research Institute, Khon Kaen University, Thailand. The research protocol was approved by the Human Research Ethics Committee, Khon Kaen University (HE571283), and informed consent was obtained from all patients before surgery.

### RNA extraction and quantitative RT-PCR

Total RNA was extracted from tissues and cell lines using Trizol reagent (Invitrogen, Carlsbad, CA, USA). The RNA concentration was quantified using a NanoDrop ND-2000 spectrophotometer (NanoDrop Technologies, Wilmington, DE, USA). The analysis of miR-210 expression was performed by the quantitative reverse transcription PCR (qRT-PCR) according to the Taqman MicroRNA Assay protocol (Applied Biosystems, Foster City, CA, USA). Briefly, one hundred nanograms of total RNA of each sample were used for cDNA synthesis using the MicroRNA Reverse Transcription kit with a specific primer (Applied Biosystems, Foster City, CA, USA). All PCR reactions were performed in duplicate on an ABI-7500 Real-Time PCR system (Applied Biosystems, Foster City, CA, USA). The miR-210 transcript abundances were normalized to the expression level of U6 small nucleolar RNA and were calculated using the 2^-ΔCt^ method.

### Cell culture

The human CCA cell lines (KKU-213, KKU-055 and KKU-100) were developed by Prof. Banchob Sripa under the Cholangiocarcinoma Research Institute, Khon Kaen University, Thailand and were obtained from JCRB Cell Bank, Japan. Cells were cultured in Ham’s F-12 medium supplemented with 10% fetal bovine serum (FBS), 100 units/ml penicillin and 100 mg/ml streptomycin. The 293T cells were cultured in Dulbecco’s modified Eagle’s medium (DMEM) supplement with 10% FBS and 1% penicillin/streptomycin. Cell culture was conducted in a humidified atmosphere containing 5% CO_2_ at 37°C. In order to induce a pseudohypoxic condition, the cells were treated with 100 μM of CoCl_2_ (Sigma-Aldrich, MO, USA).

### Plasmid constructions and viral transduction

A full-length double stranded pre-miR-210 was amplified from MCF-7 genomic DNA using PCR and cloned into a pMSCV-puro vector (Clontech, California, USA) to generate the miR-210 overexpressing pMSCV vector as described previously [22]. The miR-210 sponge vector was constructed by cloning annealed anti-miR-210 sequences into a pcDNA3.1 vector (Clontech, California, USA) and then subcloning into a lentiviral vector pLL3.7 (Clontech, California, USA) to generate a miR-210-7xSponge-pLL3.7-mCherry vector (miR-210 sponge). This vector harbors tandem repeats of anti-miR-210 sequences to inhibit miR-210 function. The reporter of the HRE (hypoxia responsive element) vector was constructed by cloning the sequences of 3xHRE (TGTCACGTCCTGCACGACTCTAGT) upstream into the pGL3 luciferase reporter vector. The HIF-1α expression vector was obtained from Addgene (plasmid number 18936). The 1.6 kb 3’-untranslated terminal region (UTR) segment of HIF-3α was amplified by PCR and inserted downstream of firefly luciferase sequences to construct pcDNA3.1-Luc-HIF-3α-3’UTR wild type as described previously [22]. The expression vector for the HIF-3α binding site mutation was generated using PCR-based methods. All expression and reporter vectors were verified by DNA sequencing.

To introduce a stable overexpression of miR-210 into CCA cells, the retroviral stocks were generated in 293T cells using pMSCV-miR-210 expression and the control vector, and subsequently transduced into KKU-213 cells. Stably miR-210 overexpressing cells were selected with 1.0 μg/ml puromycin. The expression level of miR-210 was confirmed by qRT-PCR. To perform a stable knockdown of miR-210, the miR-210 sponge vectors were transduced with a lentiviral expression vector by directly applying the viruses to the culture media of KKU-100 cells. The efficiency of transduction was estimated by visualization of DsRed (control vector) and mCherry-expressing cells (miR-210 sponge) under a fluorescence microscope and miR-210 expression level was determined by qRT-PCR.

### Transient knockdown of HIF-3α in CCA cell lines

The KKU-213 cells (1×10^5^ cells/well) were seeded in a 6-well plate. After 24 h, the HIF-3α siRNA at 50 nM final concentration (sc-38167, Santa Cruz Biotechnology) and negative control siRNA (ON-TARGETplus Non-targeting Control Pool, Dharmacon, USA) were transfected into KKU-213 cells using Lipofectamine RNAiMAX (Invitrogen) according to the manufacturer’s protocols. After 48 h of transfection, HIF-3α protein levels were examined.

### Cell proliferation assay

Cell proliferation was determined by sulforhodamine B (SRB) assay. Stably miR-210 overexpressing KKU-213 cells and stable knockdown miR-210 sponge KKU-100 cells were seeded at densities of 1.5×10^3^ cells and 3×10^3^ cells per well in a 96-well plate, respectively. At 0, 24, 48, and 72 h, cells were fixed with 10% trichloroacetic acid (Sigma-Aldrich, MO, USA). After 1 h of fixing, the cells were washed and stained with 0.4% (w/v) SRB (Sigma-Aldrich, MO, USA) for 45 min. After unbound dye had been removed by washing with 1% (v/v) acetic acid, the stained cells were solubilized with 10 mM Tris base. Absorbance was measured at 540 nm using a microtiter plate reader (Tecan Austria GmbH, Salzburg, Austria). Growth curves were constructed using mean values taken from five replicates.

### Flow cytometry analysis

Flow cytometry analysis was used to study the cell cycle status in stably overexpressing miR-210 KKU-213 and stable knockdown miR-210 sponge KKU-100 cells cultured in normoxia and CoCl_2_ to mimic hypoxia (pseudohypoxia). After CoCl_2_ treatment for 72 h, cells were trypsinized and washed with phosphate buffered saline (PBS). Cells were then fixed in 70% ethanol at 4°C overnight. After washing with PBS, cells were resuspended in PBS containing 10 μg/ml RNaseA (Qiagen, Hilden, Germany) and 30 μg/ml propidium iodide (PI) (Sigma-Aldrich, MO, USA), and incubated on ice for 30 min in the dark. Cells were then analyzed with a flow cytometer (BD Biosciences, San Jose, CA) and the different cell cycle phases were calculated using BD FACSDiva™ software (BD Biosciences, San Jose, CA).

### Clonogenic assay

CCA cells (KKU-213 and KKU-100) were seeded in 6-well plates (200 cells/well) and allowed to grow for 24 h. They were then treated with 100 μM CoCl_2_ to mimic hypoxic conditions. Cells were grown and fresh medium was provided twice a week. After 10 days of incubation, cells were fixed in 4% paraformaldehyde for 20 min and stained with 0.5% crystal violet for 10 min. The stained cells were viewed and counted under a microscope. The experiment was performed in duplicate for each condition.

### Soft agar assay

The stably transfected CCA cells (1×10^6^ cells/well) were suspended in 0.3% noble agar (Sigma-Aldrich, MO, USA) containing complete media and 20% FBS. The mixtures were then layered on a bottom agar made with 0.5% noble agar in 24-well plate. After incubation for 3–4 weeks at 37°C, the colonies were counted under a microscope.

### Luciferase reporter assay

To investigate the function of miR-210 in regulating HIF-1α activity, 10 ng of pGL3-3xHRE (Hypoxia-Response Element) reporter vector was co-transfected with 100 ng of HIF-1α expression vector, 2 μg of pcDNA3.1-miR-210-7xSponge vector or pcDNA3.1-control vector and 1 ng of Renilla vector into 293T cells using lipofectamine 2000 (Invitrogen). To confirm whether HIF-3α is a direct target of miR-210, 1 ng of pcDNA3.1-Luc-HIF3α-3’UTR wild or mutant types was co-transfected into 293T cells with 500 ng of pMSCV-miR-210 or control vector and 1 ng of Renilla vector using lipofectamine 2000. The 293T cells were grown in 24-well plates. After 48 h of transfection, firefly and Renilla luciferase activities were measured using a dual-luciferase assay (Promega) according to the manufacturer’s instructions. All reporter assays were done in triplicate.

### Western blot analysis

The whole cell lysates were prepared using RIPA lysis buffer. The 20 μg of total protein was electrophoresed in 10% sodium dodecyl sulfate-polyacrylamide gel and transferred to a polyvinylidene fluoride membrane (Whatman, Dassel, Germany). The membranes were blocked with 5% skim milk for 1 h at room temperature, followed by incubation with primary antibodies against HIF-3α (1:500, Aviva systems biology, CA, USA), HIF-1α (1:200, Abcam, Cambridge, UK) and β-actin (1:10,000, Abcam, Cambridge, UK) at 4°C overnight. After incubation with a secondary antibody, the band intensity was measured using ECL^TM^ Prime Western Blotting Detection Reagent for chemiluminescence detection (GE Healthcare UK Ltd., UK). The apparent density of the bands on membranes was captured by ImageQuant^TM^ Imager (GE Healthcare UK Ltd., UK).

### Statistical analysis

Statistical analysis was performed using SPSS 17.0 software (SPSS Inc., Chicago, IL). Levels of miR-210 in tissues were compared between normal bile ducts or adjacent non-tumorous tissues and CCA using an unpaired t-test or Mann-Whitney U-test if the data were non-parametric. The association between miR-210 levels with the patients’ clinico-pathological data was analyzed by Fisher’s exact test. A survival curve was calculated using the Kaplan-Meier method. A multivariate analysis was performed by the Cox proportional hazard regression model. Statistical comparisons between the two different groups were carried out using a Student’s t-test. A *P*-value of less than 0.05 was considered statistically significant.

## Results

### MiR-210 is abundant in human CCA tissues

**Fig 1:**
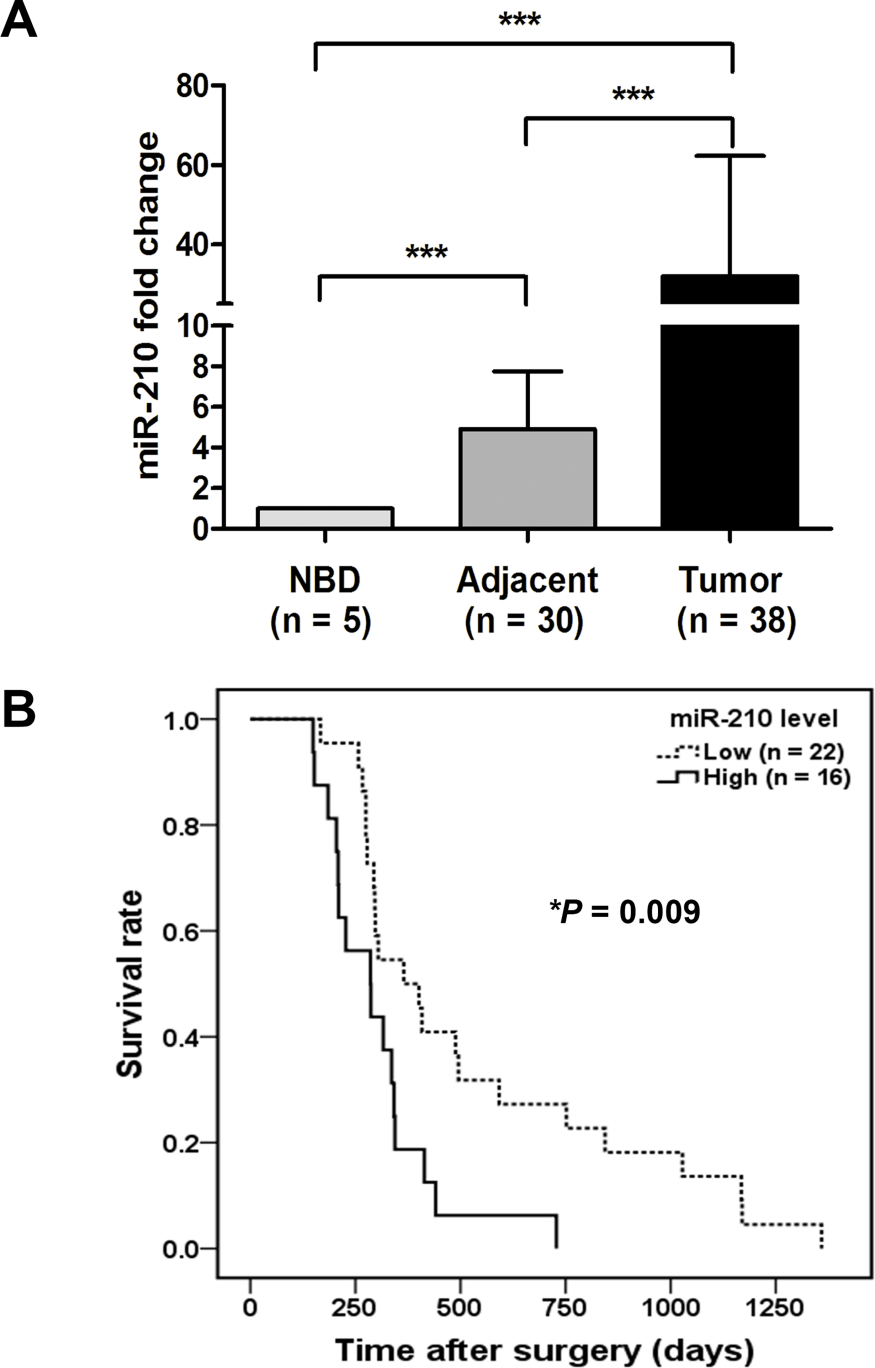
Abundance of miR-210 in CCA tumor tissues was associated with a poor prognosis. (A) The expression of miR-210 was determined in CCA tumor tissues (n = 38) compared to adjacent non-tumorous tissues (n = 30) or normal bile duct (NBD) (n = 5). Data were normalized with U6 snRNA. A Mann–Whitney *U*-test showed that miR-210 was significantly increased in tumor tissues compared with adjacent non-tumorous tissues (*P* < 0.001) and normal bile ducts (NBD) (*P* < 0.001). (B) Kaplan-Meier curves of overall survival in CCA patients showed that patients with high miR-210 expression levels (dense line; n = 16) had significantly lower survival rates than those with low miR-210 expression levels (dotted line; n = 22; *P* = 0.009).

In CCA clinical samples, the mature miR-210 expression level was significantly increased in tumor tissues compared with adjacent non-tumorous tissues or normal bile ducts (NBD) (*P* < 0.001) determined using a qRT-PCR method (Fig 1A). The association of miR-210 levels and clinico-pathological parameters was examined in CCA patients. A cut-off value was derived from the mean ± SD of the raw data for miR-210 levels in CCA tissues to separate the high (≥ 0.16) and low (< 0.16) scores. There was no correlation between miR-210 expression levels and age, gender, CCA histological type or overall metastasis. Notably however, an increased level of miR-210 was significantly associated with the shorter survival rates of the patients (*P* = 0.009, Fig 1B). A multivariate Cox regression showed that patients with a high level of miR-210 had a 2.5-fold higher risk of death than those with a low level of miR-210 in tissues (95% confidence interval [CI] 1.14–5.48, *P* = 0.02) (Table 1). These results indicate that HIF-1α responsive miR-210 is important for prognosis of CCA patients.

**Table 1:** Results of the multivariate Cox regression analysis for cholangiocarcinoma (CCA) patients’ survival.

Factors	Hazard ratio (95% CI)	*P*-value
Age <58 ≥58	1 (Reference) 1.02 [0.50–2.09]	0.951
Sex Female Male	1 (Reference) 0.62 [0.28–1.37]	0.239
CCA type Non-papillary type Papillary type	1 (Reference) 0.86 [0.39–1.88]	0.701
Overall metastasis Negative Positive	1 (Reference) 1.92 [0.92–3.99]	0.081
MiR-210 expression level Low High	1 (Reference) 2.51 [1.14–5.48]	0.022*

*P-value less than 0.05 was considered statistically significant.

### HIF-1α-responsive miR-210 in CCA cell lines

**Fig 2:**
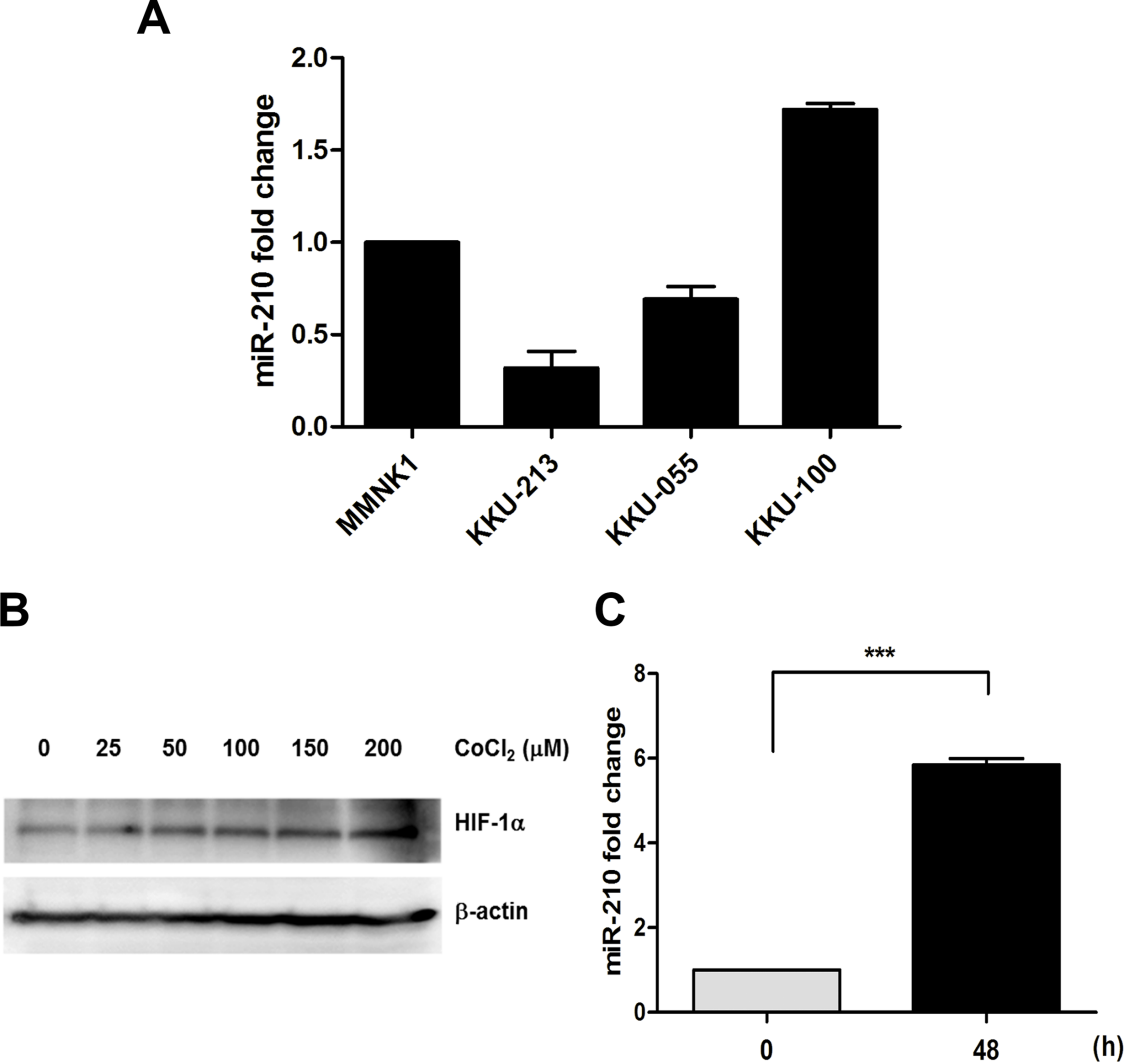
CoCl_2_ treatment stabilizes HIF-1α and induces miR-210 expression in CCA cells. (A) The expression levels of HIF-1α in CCA cell lines (KKU-213, KKU-055 and KKU-100). (B) HIF-1α expression level in KKU-213 cells treated with 0, 25, 50, 100, 150 and 200 μM of CoCl_2_ for 48 h. (C) The miR-210 expression levels in KKU-213 cells treated with 100 μM CoCl_2_ at 0 and 48 h. ***P < 0.001.

We examined the expression levels of miR-210 in immortalized cholangiocyte cells (MMNK1) and CCA cell lines (KKU-213, KKU-055 and KKU-100). Based on the variable expression pattern (Fig 2A), we chose KKU-213 (low miR-210) and KKU-100 (high miR-210) for further studies. CoCl_2_ treatment stabilized the hypoxic condition by inhibiting a key enzyme prolyl hydroxylase resulting in prevention of HIF-1α being degraded by the ubiquitin-proteasome pathway [23]. Tumor cells under the CoCl_2_ treatment accelerated their metastatic ability [21,24] and enhanced chemoresistance to 5-fluorouracil and oxaliplatin [13]. As presented in Fig 2, KKU-213 cells were treated with CoCl_2_ for 48 h. We found that the HIF-1α protein levels were increased in response to CoCl_2_ treatment in a dose dependent manner (Fig 2B). The miR-210 level was increased 5.8-fold when treated with 100 μM CoCl_2_ (Fig 2C). Similar results were observed in KKU-100 cells (S1 Fig), indicating that miR-210 was induced under pseudohypoxia in the CCA cells.

### MiR-210 inhibits CCA cell proliferation

**Fig 3:**
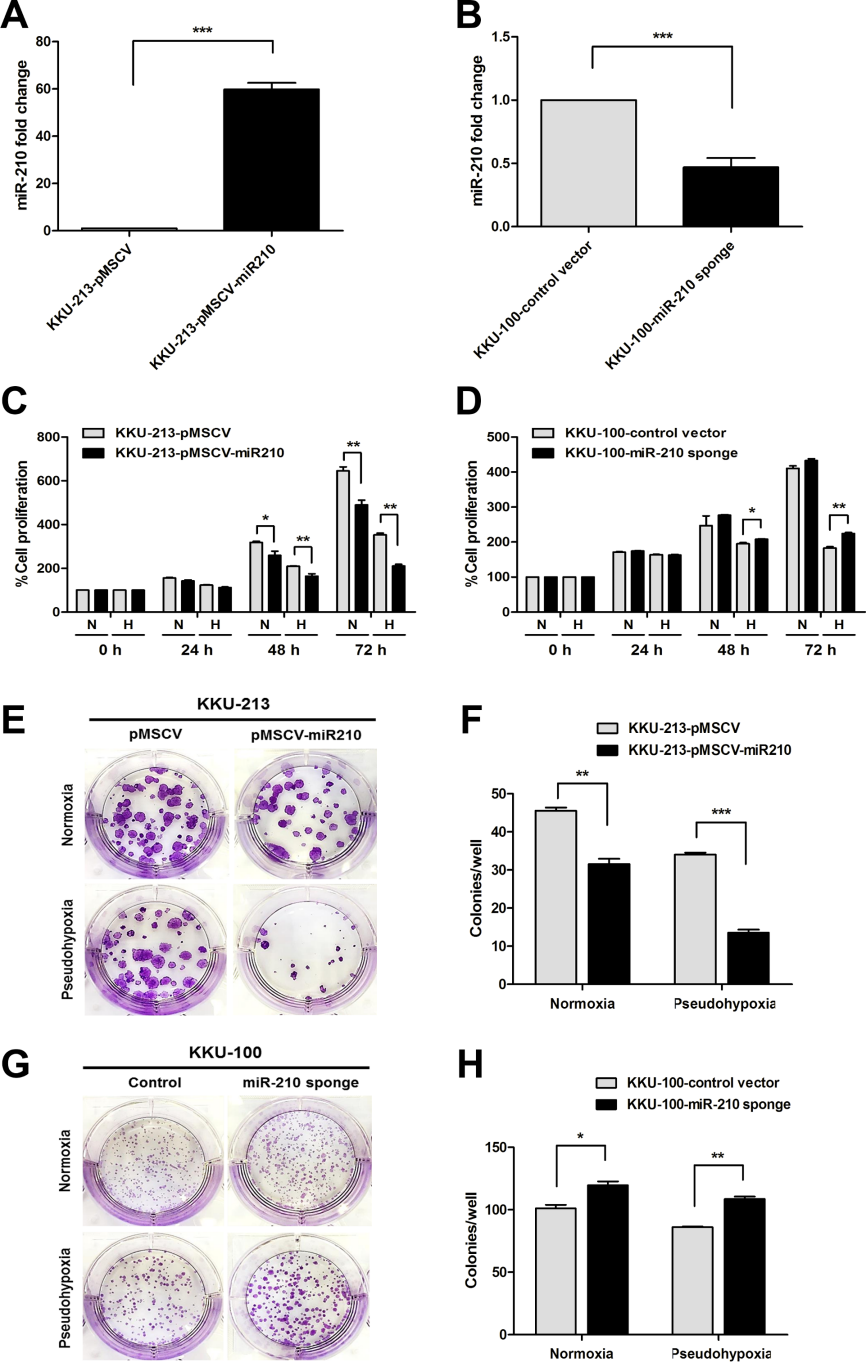
MiR-210 inhibits cell proliferation in CoCl_2_-induced pseudohypoxia. (A) The expression level of miR-210 was detected in the stably miR-210 overexpressing KKU-213 and (B) in the stable knockdown miR-210 sponge KKU-100 cells compared to control cells. (C) A cell proliferation assay was done in stable KKU-213 cell cultured in normoxia (N) and pseudohypoxia (H). (D) A cell proliferation assay was done in stable KKU-100 cells cultured in normoxia and pseudohypoxia. The results for absorbance values at 540 nm are presented as mean ± SD. (E, F) A clonogenic assay was performed in stably overexpressing KKU-213 cells and their control cultured in normoxia (upper panel) and pseudohypoxia (lower panel) for 10 days. (G, H) A clonogenic assay was performed in stable knockdown KKU-100 cells and their control cultured in normoxia (upper panel) and pseudohypoxia (lower panel) for 14 days. *P < 0.05. **P < 0.01. ***P < 0.001.

To determine how miR-210 functions in responding to pseudohypoxic conditions *in vitro*, miR-210 expressing plasmid and its control vector were transduced into KKU-213 cells in order to produce stably overexpressing miR-210 cells. Moreover, miR-210 sponge plasmid (the miR-210 anti-sense strand) and its control vector were transduced into KKU-100 cells in order to construct a stable knockdown of miR-210 cells. The results obtained from qRT-PCR demonstrated that the stably miR-210 overexpressing KKU-213 cells showed a 59-fold increase in miR-210 expression levels over the control cells (Fig 3A). The stable knockdown miR-210 sponge KKU-100 cells decreased by 53% of the endogenous miR-210 levels over the control cells (Fig 3B). The modest decrease by miR-210 sponge compared to increase by overexpression was probably due to highly abundant nature of endogenous miR-210 (miRBase, http://www.mirbase.org/) [25].

The role of miR-210 on cell proliferation demonstrated by SRB assay was examined in both stably transduced CCA cell lines cultured in normoxic and pseudohypoxic conditions after 72 h incubation. The stably miR-210 overexpressing KKU-213 cells showed a significant decrease in cell proliferation compared with the control cells in both normoxia and pseudohypoxia (*P* < 0.01, Fig 3C). We examined cell proliferation in the stable knockdown miR-210 sponge KKU-100 cells, and their cell proliferation showed no significant difference compared with control cells under normoxic conditions. In contrast, the use of knockdown miR-210 sponge in KKU-100 cells significantly increased cell proliferation under pseudohypoxia (*P* < 0.01, Fig 3D). To confirm CoCl_2_ pseudohypoxia, we placed miR-210 sponge KKU-100 cells into a hypoxia chamber (0.5%O_2_). The results obtained under hypoxia chamber conditions were consistent with those obtained using CoCl_2_ pseudohypoxic conditions (S2 Fig).

A clonogenic assay was used to determine tumor cell growth *in vitro* after an incubation period of 14 days to allow colony formation. The stably miR-210 overexpressing KKU-213 cells showed markedly decreased colony formation compared with control cells cultured in both normoxia (*P* = 0.003) and pseudohypoxia (*P* < 0.001, Fig 3E and 3F). On the other hand, the depletion of endogenous miR-210 in KKU-100 cells showed significantly increased colony formation compared with control cells cultured in both normoxia (*P* = 0.013) and pseudohypoxia (*P* = 0.005, Fig 3G and 3H).

### MiR-210 increased cell cycle arrest at G2/M phase under CoCl_2_-induced pseudohypoxia

**Fig 4:**
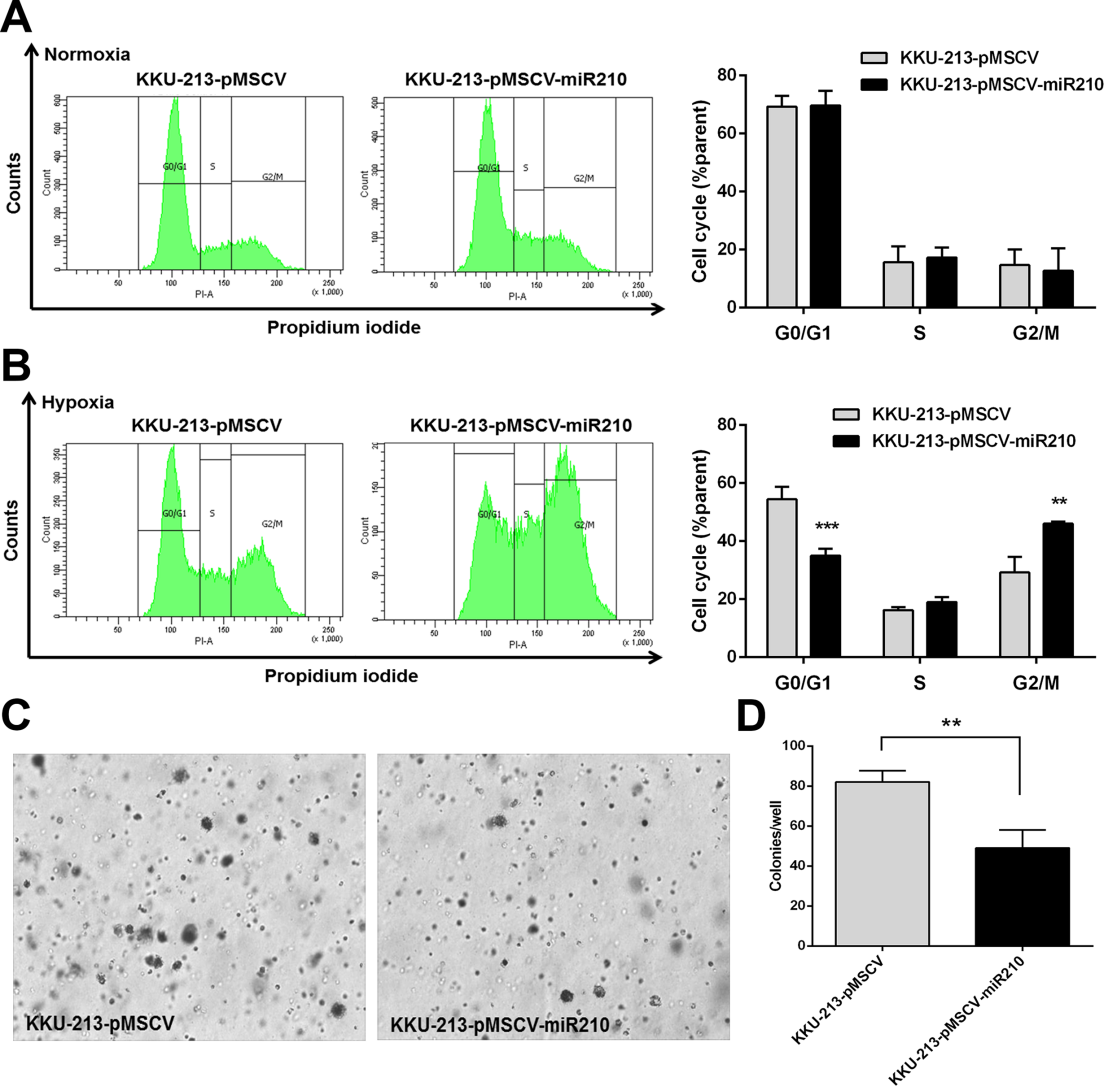
Alterations in the cell cycle in miR-210 overexpressing cells cultured in CoCl_2_-induced pseudohypoxia. Stably overexpressing KKU-213 cells were cultured under normoxia and in 100 mM CoCl_2_ for 72 h, respectively. The graphs in Fig 4A and 4B show cell proportions in different phases of the cell cycle in miR-210 overexpression cells compared to control cells. The data present the mean ± SD of three-independent experiments. (C, D) Soft agar assay results in KKU-213 cells cultured under normoxia for 30 days. **P < 0.01. ***P < 0.001.

To determine the mechanism by which miR-210 suppressed CCA cell growth, cell cycle analysis was performed using flow cytometry. Stably transfected CCA cells were cultured under normoxia and pseudohypoxia for 72 h and the cell cycle was analyzed. There was no significant change in cell proportions between the miR-210 overexpressing cells *versus* control cells cultured under normoxia at all phases, including G0/G1 (69±5.1% and 69±3.7%), S (17±3.4% and 16±5.5%), and G2/M (13±7.8% and 15±5.4%), respectively (Fig 4A). In contrast, the miR-210 overexpressing cells cultured under pseudohypoxia showed a significant decrease in the proportion of cells at the G0/G1 phase when compared with control cells (35±2.4% and 54±4.3, *P* < 0.001). In Fig 4B, miR-210 overexpressing cells cultured in pseudohypoxia showed a significant increase in proportion of cells at the G2/M phases compared with control cells (46±0.8% and 29±5.4%, *P* = 0.004). In addition, the soft agar assay revealed that the miR-210 overexpressing KKU-213 cells were significantly retarded in colony formation under pseudohypoxia (*P* = 0.006, Fig 4C and 4D).

### MiR-210 diminished gemcitabine sensitivity under CoCl_2_-induced pseudohypoxia

**Fig 5:**
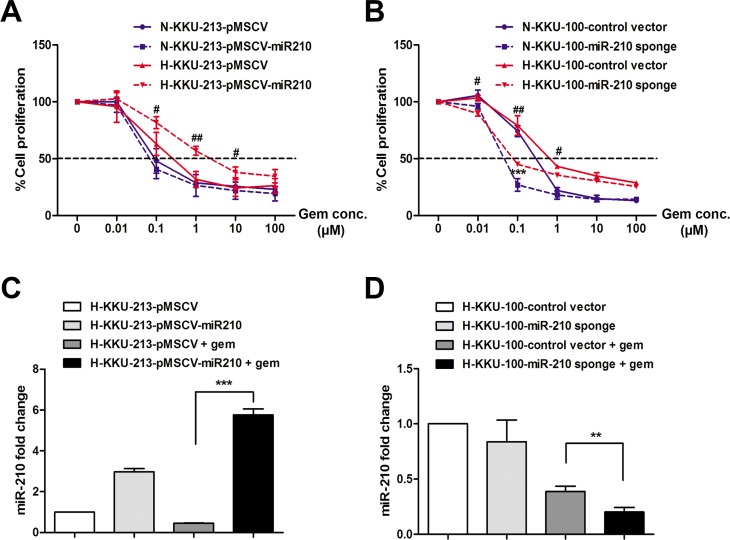
MiR-210 diminished gemcitabine sensitivity under CoCl_2_-induced pseudohypoxia. (A) The stably miR-210 overexpressing KKU-213 cells were incubated with gemcitabine and cultured under normoxia (N) or pseudohypoxia (H) for 72 h. (B) The stable knockdown miR-210 sponge KKU-100 cells were incubated with gemcitabine and cultured under normoxia (N) and pseudohypoxia (H) for 72 h. (C) The fold change of miR-210 expression in the stably miR-210 overexpressing KKU-213 cells incubated with 4 μM gemcitabine under pseudohypoxia. (D) The fold change of miR-210 expression in the stable knockdown miR-210 sponge KKU-100 cells incubated with 1 μM gemcitabine under pseudohypoxia. The data present the mean ± SD of three-independent experiments. *P < 0.05. **P < 0.01. ***P < 0.001.

To investigate the effect of miR-210 on the drug sensitivity of CCA cells, the stably miR-210 overexpressing KKU-213 cells and the stable knockdown miR-210 sponge KKU-100 cells were incubated with the cytotoxic drug gemcitabine and cultured under normoxia and pseudohypoxia for 72 h. As shown in Fig 5A, the IC_50_ values of stably miR-210 overexpressing KKU-213 cells showed no difference compared with control cells cultured under normoxic conditions (0.09±0.01 and 0.10±0.02 μM, respectively), whereas stably miR-210 overexpressing KKU-213 cells cultured under pseudohypoxia showed a significant increase in IC_50_ values toward gemcitabine compared with the control cells with a sensitivity index (SI) of 0.12 (3.58±1.23 and 0.42±0.21 μM, respectively, *P* = 0.013). For the knockdown experiment, the stable miR-210 sponge KKU-100 cells showed a significant decrease in IC_50_ values against gemcitabine compared with control cells under both normoxia (0.07±0.01 and 0.46±0.05 μM, respectively, *P* < 0.001, Fig 5B) and pseudohypoxia (0.09±0.01 and 0.81±0.07, respectively, *P* = 0.003). The stable knockdown miR-210 sponge KKU-100 cells depleting endogenous miR-210 showed an increase in sensitivity to gemcitabine with a sensitivity index of 7.03 when cultured under normoxia and 9.26 when cultured under pseudohypoxia (Table 2). In addition, to determine the expression level of miR-210 in responding to gemcitabine treatment under pseudohypoxia, the stably miR-210 overexpressing KKU-213 cells and the stable knockdown miR-210 sponge KKU-100 cells were incubated with gemcitabine under pseudohypoxia for 72 h. The results showed that miR-210 was maintained at the high level to reciprocate to gemcitabine treatment in miR-210 overexpressing KKU-213 cells compared with control cells (Fig 5C, *P* < 0.001). The level of miR-210 was suppressed in the miR-210 knockdown KKU-100 cells under pseudohypoxic conditions in the present of gemcitabine compared with the control (Fig 5D, *P* < 0.01). These results indicate that miR-210 is involved in the drug response and overexpression of miR-210 under pseudohypoxia diminished gemcitabine sensitivity in CCA cells.

**Table 2:** IC_50_ value and sensitivity index in stable CCA cell lines to gemcitabine.

Conditions	IC_50_ value of gemcitabine (μM)					
	Normoxia	SI	*P*-value	Hypoxia	SI	*P*-value
KKU-213-pMSCV	0.10±0.02			0.42±0.21		
KKU-213-pMSCV-miR210	0.09±0.01	1.12	0.257	3.58±1.23	0.12	0.013*
KKU-100-control vector	0.46±0.05			0.81±0.07		
KKU-100-miR210 sponge	0.07±0.01	7.03	<0.001*	0.09±0.01	9.26	0.003*

SI, sensitivity index.

*P-value less than 0.05 was considered statistically significant.

### HIF-3α identified as a miR-210 target

**Fig 6:**
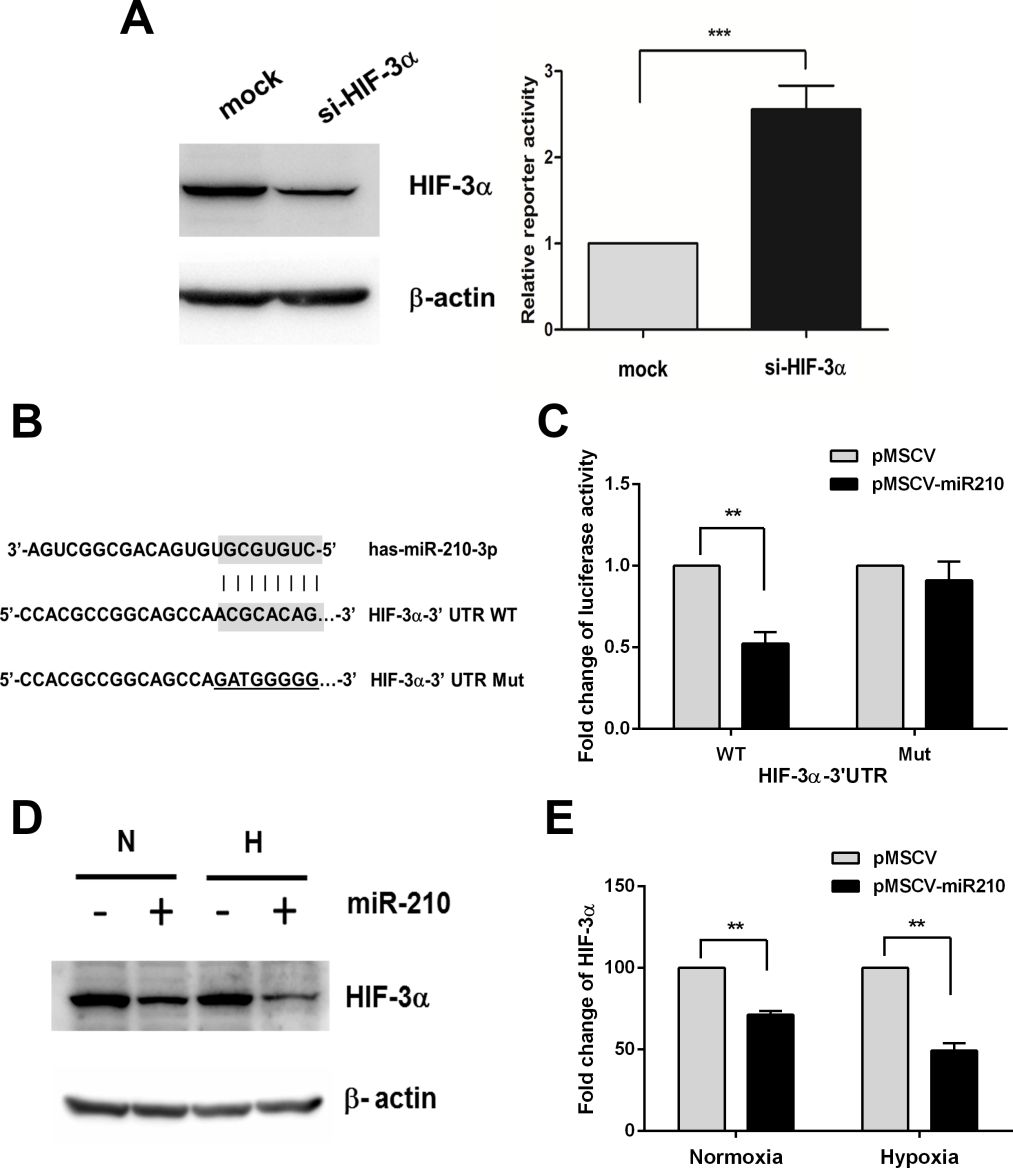
MiR-210 mediates HIF-1α activity though targeting HIF-3α. (A) HRE (hypoxia responsive element)-reporter assay in KKU-213 cells KKU-213 cells transiently transfected with 50 nM of HIF-3α siRNA compared with control cells (mock). (B) The *in silico* prediction using TargetScan database demonstrated the miR-210 binding site on 3’UTR of HIF-3α in both wild-type and mutant forms. (C) The 293T cells were transfected with Luc-HIF-3α-3’UTR wild-type or mutant reporter vector as well as miR-210 expression and control vectors. After 72 h transfection, the cells were lysed and the luciferase activity was detected. Data are presented as the luciferase activity normalized with the luciferase activity of Renilla. (D, E) Protein level of HIF-3α in the miR-210 overexpressing cells cultured in normoxia (N) and pseudohypoxia (H). The data present the mean ± SD of three-independent experiments. **P < 0.01. ***P < 0.001.

As miR-210 contributed to the suppression of CCA cell proliferation, but induced drug resistance, we hypothesized that miR-210 is augmenting HIF-1α activity. To determine the effect of HIF-3α knockdown on the HIF-1α function, we performed the HRE-reporter assay to assess the HIF-1α activity. Based on previous observations and *in silico* analysis (TargetScan), we found that HIF-3α is a potential target of miR-210, and HIF-3α functions as negative regulator of HIF-1 transcription factor [26,27]. Consistently, knockdown of HIF-3α increased HRE-reporter activity of HIF-1α (Fig 6A). To identify whether HIF-3α is directly targeted by miR-210, the wild-type and mutant forms of HIF-3α 3’UTR reporter were constructed (Fig 6B). We demonstrated that the HIF-3α 3’UTR wild-type form showed a significant decrease in luciferase activity of up to 50% by miR-210, but this effect was not observed for the mutant form compared to the controls (*P* = 0.017, Fig 6C). In addition, western blot analysis showed that the overexpression of miR-210 in KKU-213 cells significantly suppressed HIF-3α protein expression in both normoxia and pseudohypoxia (*P* < 0.01, Fig 6D and 6E). These results suggest that miR-210 regulates HIF-1α activity via targeting of HIF-3α.

### miR-210 constitutes a regulatory loop of HIF-1α activity

**Fig 7:**
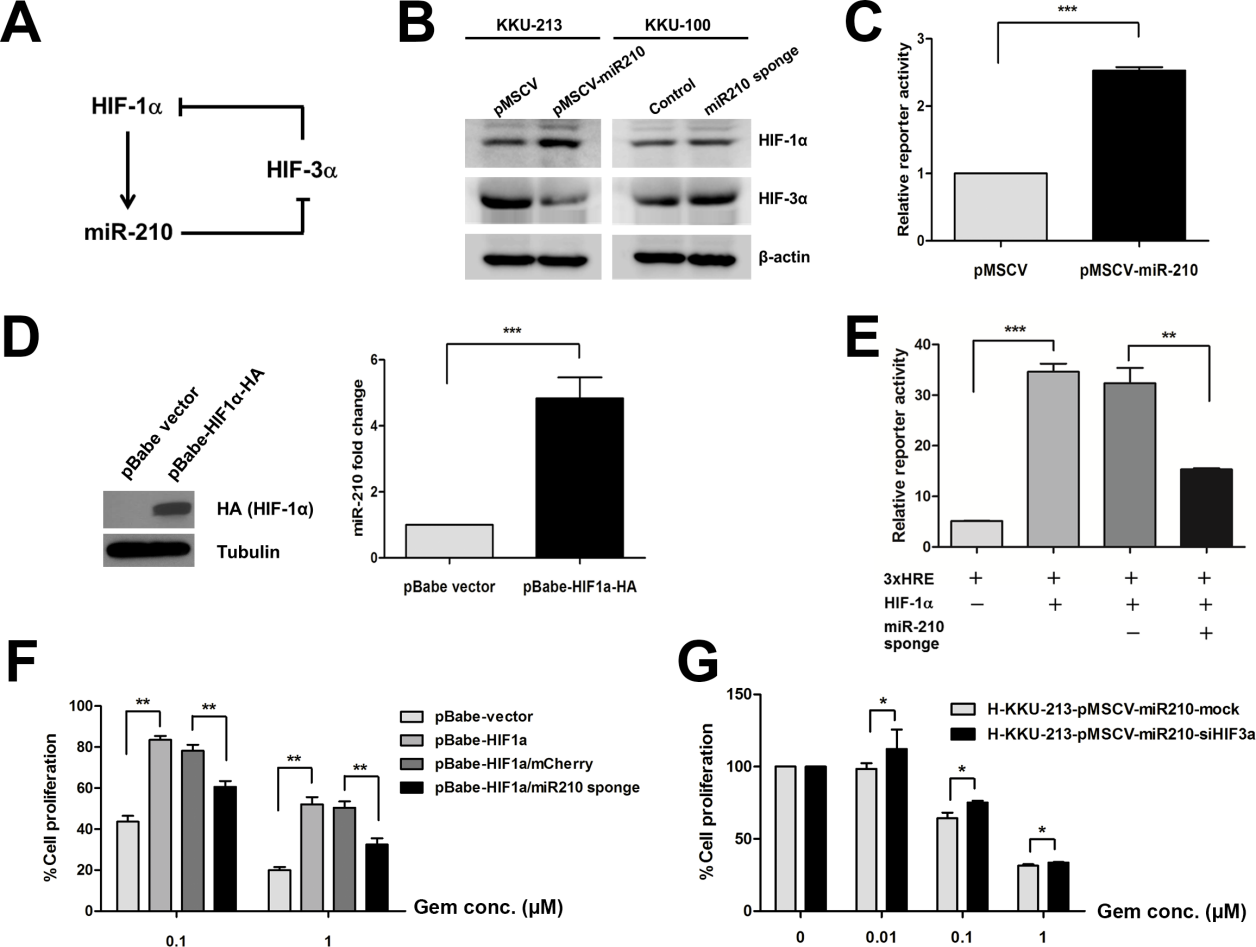
Suppression of miR-210 regulates HIF-1α activity *via* the function of HIF-3α. (A) A schematic diagram shows the hypothetical role of miR-210 in regulating HIF-1α function. (B) Protein levels of HIF-1α and HIF-3α in the stably transfected CCA cells. (C) HRE (hypoxia responsive element)-reporter assay in KKU-213 cells induced by miR-210 expression vector. (D) The expression level of miR-210 in KKU-213 cells transiently overexpressed with HIF-1α vector. (E) The 293T cells were co-transfected with Luc-3xHRE, HIF-1α expression vector and miR-210 sponge as well as control vectors. At 72 h post-transfection, cells were lysed and luciferase activity was determined. Data were normalized with the luciferase activity of Renilla. (F) Cell proliferation assay in KKU-213 cells transiently transfected with HIF-1α and miR-210 sponge vector in responding to gemcitabine treatment. (G) Cell proliferation assay in the stable miR-210 overexpressing KKU-213 treated with si-HIF-3α and incubated with gemcitabine under pseudohypoxia for 72 h. The data present the mean ± SD of three-independent experiments. *P < 0.05. **P < 0.01. ***P < 0.001.

Based on the observation that HIF-1α responsive miR-210 is a target for HIF-3α, we hypothesized that miR-210 constitutes a HIF-1α regulatory loop *via* targeting HIF-3α as shown in a schematic diagram (Fig 7A). To prove this hypothesis, we determined the expression level of HIF-1α and HIF-3α in the stably CCA cells. In Fig 7B, the results showed that HIF-1α was increased in the stably miR-210 overexpressing KKU-213 cells whereas HIF-3α was decreased. On the other hand, the expression level of HIF-3α was increased in the stable knockdown miR-210 sponge KKU-100 cells (Fig 7B). Consistently, introduction of miR-210 in KKU-213 cells increase HIF-1α transcriptional activity as determined by HRE-reporter (Fig 7C). To re-validate HIF-1α-dependent miR-210 expression, we overexpressed HA-tagged HIF-1α expression vector in KKU-213 cells and measured mature miR-210 abundance. Consistent with previous observations [16], miR-210 abundance was significantly increased by expression of HIF-1α (Fig 7D). Moreover, knockdown of HIF-1α led to decreasing of miR-210 expression level resulting in blocking the effect of miR-210 overexpression on the decrease of cell proliferation in pseudohypoxic condition (S3 Fig), supporting that miR-210 is induced by HIF-1α in CCA cells. To examine the regulatory loop consists of miR-210 and HIF-3α on HIF-1α function, we depleted endogenous miR-210 with the miR-sponge vector and examined the HIF-1α transcriptional function. The luciferase reporter assay demonstrated that cells transfected with the HIF-1α expression vector showed a significant increase in luciferase activity due to the binding of HIF-1α to 3xHRE. In contrast, co-transfection of the HIF-1α expression vector and the miR-210 sponges into 293T cells showed a significant decrease in luciferase activity compared with the controls (*P* < 0.05, Fig 7E). Our results suggested that suppression of miR-210 inhibits the activity of HIF-1α *via* binding of HIF-3α, which is proposed as a negative control of HIF-1α. Despite the high aggression of CCA, the therapeutic options for CCA are limited. Gemcitabine is one of the most widely used approved drugs for CCA patients and the high HIF-1α activity reduces sensitivity to gemcitabine [28,29]. Given the miR-210-mediated gemcitabine sensitivity in CCA cells, we finally examined the functional relevance of miR-210/HIF-3α regulatory loop on gemcitabine sensitivity. HIF-1α overexpression in KKU-213 cells treated with gemcitabine increased the cell proliferation, while the depletion of miR-210 with the sponge vector suppressed the CCA cell proliferation under the gemcitabine treatment (*P* < 0.01, Fig 7F). The stably miR-210 overexpressing KKU-213 cells were transfected with 50 nM si-HIF-3α and then treated with gemcitabine (0, 0.01, 0.1 and 1 μM) under pseudohypoxic condition for 72 h. The result showed the increase of percentage of cell proliferation in the stably miR-210 overexpressing KKU-213 cells with HIF-3α suppression compared with the control (*P* < 0.05, Fig 7G). Therefore, miR-210/HIF-3α regulatory axis possibly constitutes a regulatory loop of HIF-1α activity in CCA cells.

## Discussion

The upregulation of miR-210, a major hypoxia-responsive miRNA, has been reported in most solid tumors, and high levels are correlated with a poor clinical outcome for patients [30–32]. In this study, we demonstrated that miR-210 was significantly increased in CCA tumor tissues and was associated with shorter survival of patients, suggesting that it possibly serves as an independent indicator to predict the patients’ prognosis. The fact that a high miR-210 expression level is associated with poor clinical outcomes has been found in several other cancers, such as head and neck cancer [30], soft-tissue sarcoma [33], glioblastoma [34], renal cell carcinoma [35] and breast cancer [16,32]. The study of intrahepatic CCA shows that HIF-1α presents in CCA tissues with 50% of all cases showing high expression scores; thus it indicated that the hypoxic state is activated in CCA [21]. In addition, it has been demonstrated that HIF-1α recognizes the hypoxia responsive element (HRE) on the miR-210 promoter [15]. This induction by HIF-1α expression is perhaps associated with the miR-210 levels in CCA.

Our data revealed that overexpression of miR-210 strongly inhibited cell proliferation, whereas knockdown of miR-210 significantly increased cell proliferation. Consensus results were clearly obtained from the colony formation assay, which took a longer period of time for tumor cells growth than the SRB assay. The correlation between miR-210 and cell proliferation has been studied in many cancers and diseases. Our observation is consistent with earlier reports that suggest the overexpression of miR-210 inhibits cell proliferation [36–38]. However, several other studies reported that miR-210 promoted cell proliferation [39–42]. The diverse roles of miR-210 may be due to different interactions with different targets and cell types.

Interestingly, our study found that overexpression of miR-210 induced cell cycle arrest at the G2/M phase under CoCl_2_-induced pseudohypoxia, leading to diminished gemcitabine sensitivity in CCA cell lines. The gemcitabine resistance in CCA cells was previously studied by Wattanawongdon *et al*., who demonstrated that the acquired gemcitabine-resistant cells changed their cell cycle distributions by increasing the proportions in the S and G2/M phases [43]. In addition, HeLa cells were treated with doxorubicin or cisplatinum showing that their subpopulations going through mitosis subsequently underwent apoptosis, while the cells arrested in S/G2/M survived [44]. Moreover, cell cycle-mediated drug resistance has been reported for taxanes, which stabilized cytoplasmic microtubules and block cells in the G2/M phase of the cell cycle [45]. Thus, the result from knockdown of miR-210 by miR-210 sponges inducing CCA cell growth facilitates CCA cells to become more sensitive to gemcitabine. The upregulation of miR-210 is correlated with a poor prognosis in many solid tumors, suggesting that miR-210 and its target genes have functional influences on cancer progression and drug resistance. It is noted that there are 38 cases of patients’ tissues included in this study but only 12 patients have a history of gemcitabine therapy, therefore it’s not valid for the statistical analysis. Due to gemcitabine sensitivity according to miR-210 constitutes a separate clinical study, we plan to collect the cases recruited in the clinical trials of gemcitabine that is an ongoing project. In addition, the patients’ samples presented in Fig 1 were collected from the CCA patients without pretreatment, such as chemotherapy or radiotherapy. Recently, it has been reported that a combination of knockdown miR-210 with radiotherapy enhanced the anti-tumor effect on human hepatoma xenografts [46] Therefore, targeting miR-210 may enhance the effectiveness of cancer treatment. Overexpression of miR-210 in CCA tissues might help predict chemosensitivity to gemcitabine, and this data will provide useful information for current clinical trials in our CCA patients.

An essential step towards understanding the function of miR-210 is identifying its relevant targets. Several genes have been experimentally identified as miR-210 targets responding to hypoxic environments [47]. We show that HIF-3α is the direct target of miR-210 as demonstrated in 293T cells. Our study in CCA cells confirmed that overexpression of miR-210 suppressed the protein level of HIF-3α in KKU-213 CCA cells. Further, suppression of HIF-3α by siRNA leads to the attenuation of CCA cell proliferation. Our findings indicate that miR-210 regulates CCA growth *via* HIF-3α activity. The role of HIF-3α in controlling cell growth was suspected in a previous study demonstrating that knockdown of HIF-3α retards endothelial cell growth in both normoxic and pseudohypoxic conditions [48]. However, the study from Li *et al*. demonstrated that knockdown of HIF-3α promoted an abnormal proliferation in osteoarthritis. Targeting of HIF-3α by miR-210 results in increasing chondrocyte proliferation and prompts the extracellular matrix deposition on osteoarthritis [49]. Thus, the role of miR-210 on targeting HIF-3α may have diverse effects in various cell types.

A positive feedback loop between miR-210 and HIF-1α is crucial for cell survival under hypoxia [50]. We hypothesized that miR-210 mediated HIF-1α signaling through targeting its negative regulators. It has been reported that HIF-3α is a negative regulator of HIF-1α and can inhibit HIF-1α activity [26,27]. In this study, we identified HIF-3α, a negative regulator of HIF-1α, as a direct target of miR-210. Suppression of miR-210 diminished HIF-1α activity. The study in hepatocellular carcinoma demonstrated that the HIF-1α/miR-210/HIF-3α feedback circuit plays a regulatory role in TIMP2 suppression [51]. Taken together, it is likely that miR-210 modulates HIF-1α by targeting HIF-3α. Nevertheless, the molecular linkage between HIF-1α, miR-210 and its target HIF-3α in CCA cells needs to be further investigated.

In conclusion, the present study demonstrates that miR-210 regulates HIF-1α signaling by targeting HIF-3α, leading to stabilization of the activity of HIF-1α. The expression of HIF-3α is crucial for responding to hypoxia by a negative feedback loop to maintain balance under hypoxic conditions. Overexpression of miR-210 in cancer cells may contribute to a persistent function of HIF-1α by regulating HIF-3α and other targets, leading to the progression of the cancer by attenuating cell proliferation and increasing drug resistance. The high expression of miR-210 may be involved in the progression of cancer and can possibly be a potential indicator for the efficacy of gemcitabine in CCA therapy.

## Supporting information

S1 Fig CoCl_2_ treatment stabilizes HIF-1α and induces miR-210 expression in KKU-100 cells. (A) HIF-1α expression level in KKU-100 cells treated with 0, 25, 50, 100, 150 and 200 μM of CoCl_2_ for 48 h. (B) The fold change of miR-210 expression levels in KKU-100 cells treated with 100 μM CoCl_2_ at 0, 12 and 24 h.
(TIF)

S2 Fig Cell proliferation in KKU-100 cultured in a hypoxia chamber. The stably miR-210 sponge KKU-100 cells were cultured in a hypoxia chamber (0.5%O_2_) for 72h. The cell proliferation was performed using SRB assay. Data were presented as mean ± SD. *P < 0.05.
(TIF)

S3 Fig The knockdown of HIF-1μ in the miR-210 overexpressing KKU-213 cells. Cells were treated with 100 nM si-HIF-1α for 72 h and investigated for HIF-1α and HIF-3α expression levels (A), miR-210 level (B), and clonogenic assay (C). ***P < 0.001.
(TIF)
