# Supplementary figures and images for: Potential role of HIF-1-responsive microRNA210/HIF3 axis on gemcitabine resistance in cholangiocarcinoma cells
Source: PLoS One. 2018 Jun 28;13(6):e0199827. doi: 10.1371/journal.pone.0199827 (PMC6023215; doi:10.1371/journal.pone.0199827)

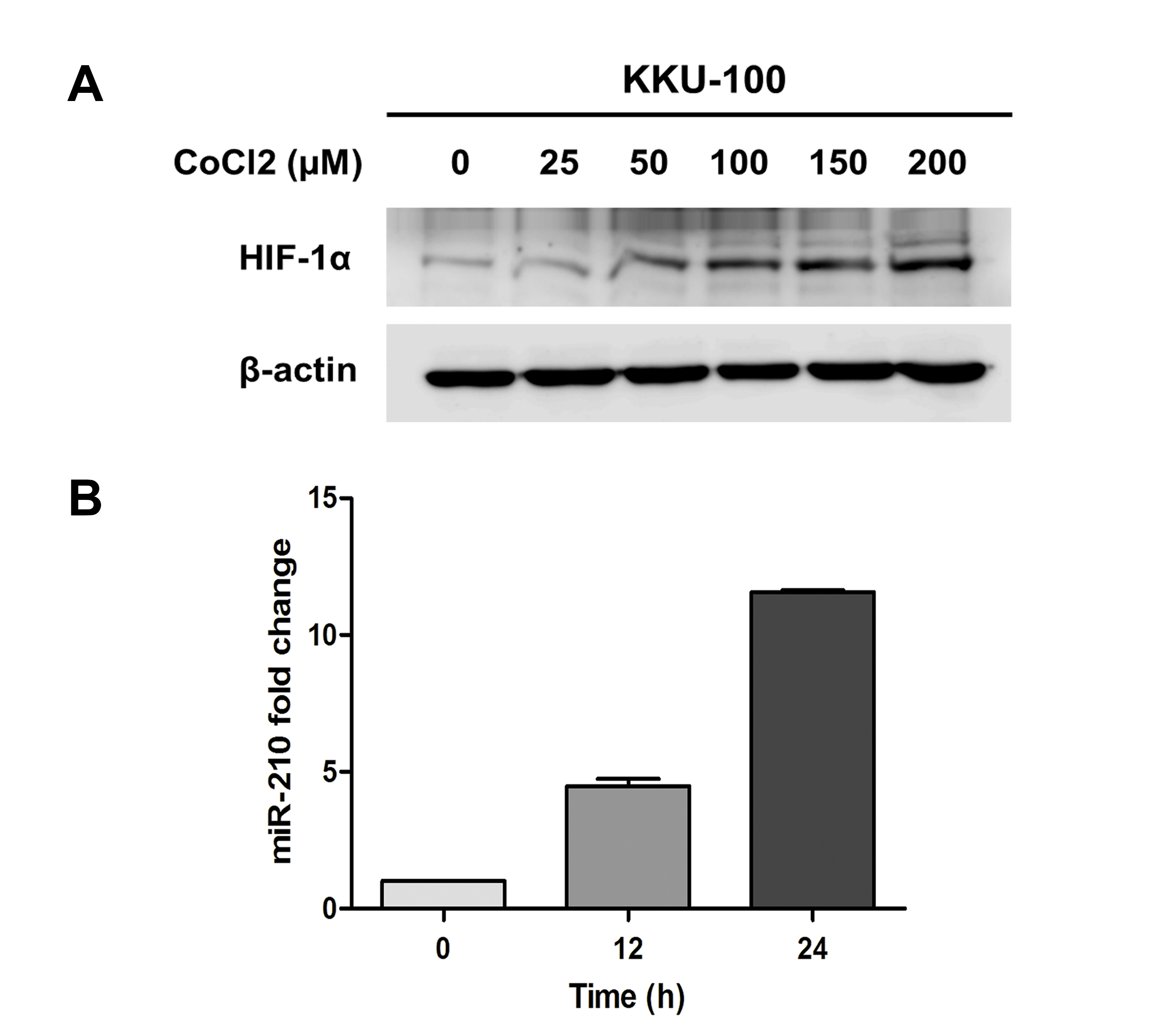

Supplement: S1 Fig — (A) HIF-1α expression level in KKU-100 cells treated with 0, 25, 50, 100, 150 and 200 μM of CoCl2 for 48 h. (B) The fold change of miR-210 expression levels in KKU-100 cells treated with 100 μM CoCl2 at 0, 12 and 24 h. (TIF) [file pone.0199827.s001.tif]

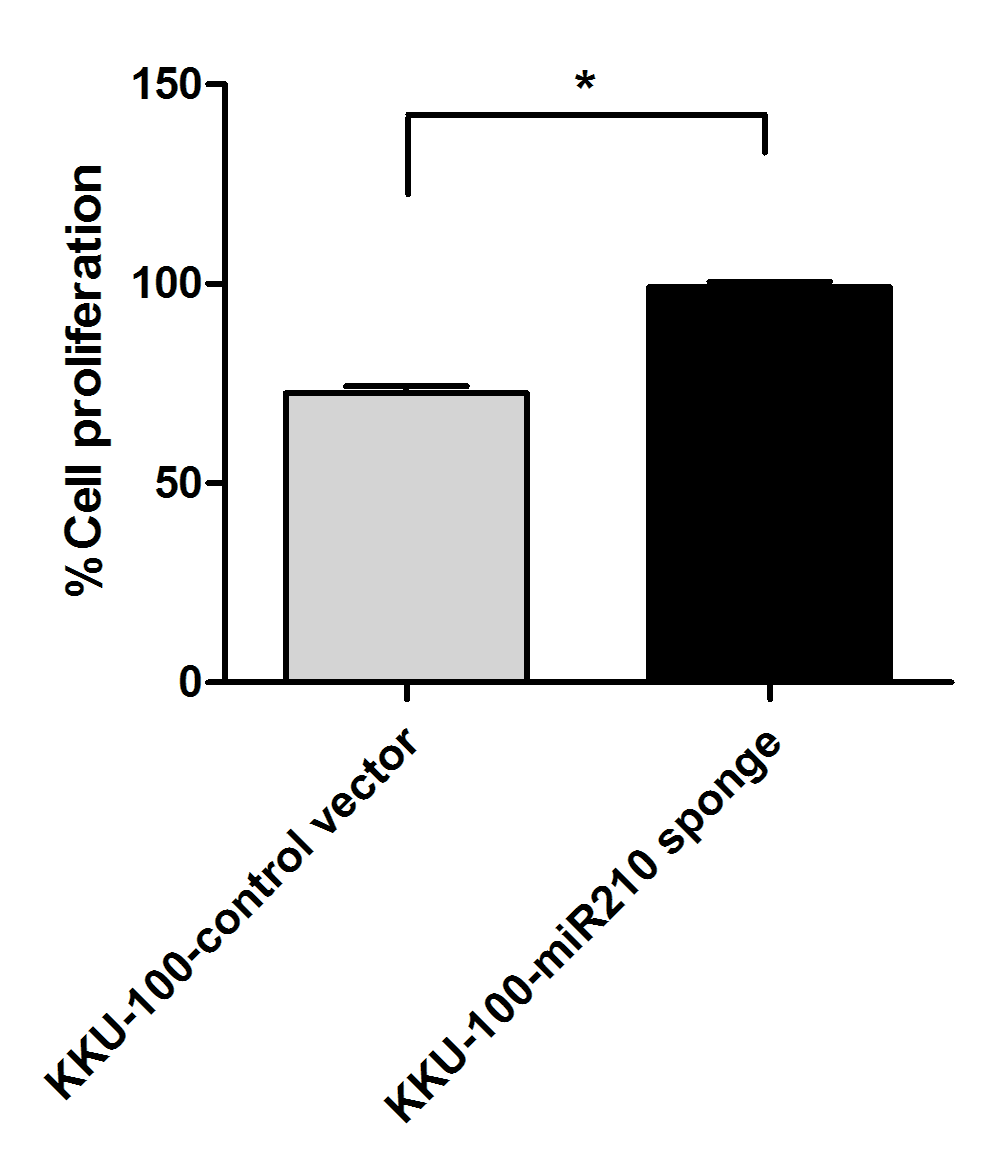

Supplement: S2 Fig — The stably miR-210 sponge KKU-100 cells were cultured in a hypoxia chamber (0.5%O2) for 72h. The cell proliferation was performed using SRB assay. Data were presented as mean ± SD. *P < 0.05. (TIF) [file pone.0199827.s002.tif]

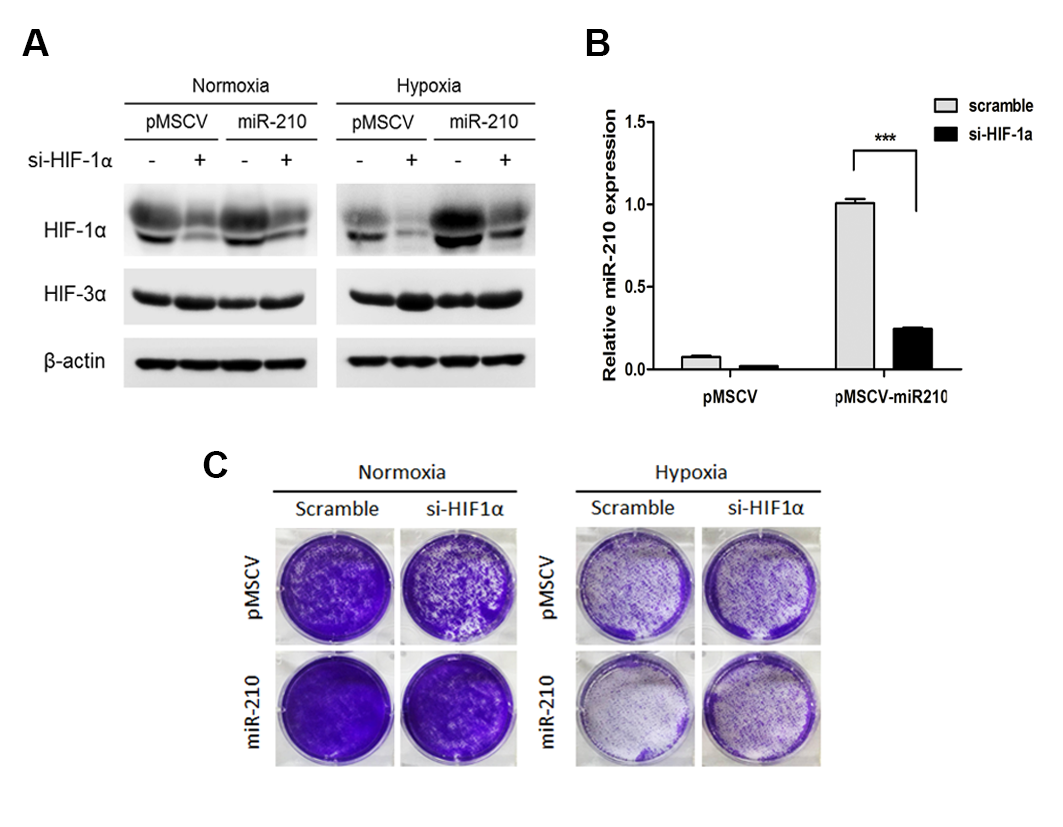

Supplement: S3 Fig — Cells were treated with 100 nM si-HIF-1α for 72 h and investigated for HIF-1α and HIF-3α expression levels (A), miR-210 level (B), and clonogenic assay (C). ***P < 0.001. (TIF) [file pone.0199827.s003.tif]
